# Some Features of the Ultrasonic Liquid Extraction of Metal Ions

**DOI:** 10.3390/molecules24193549

**Published:** 2019-09-30

**Authors:** O.M. Gradov, Yu.A. Zakhodyaeva, I.V. Zinov’eva, A.A. Voshkin

**Affiliations:** Kurnakov Institute of General and Inorganic Chemistry, Russian Academy of Sciences, 31 Leninsky Prospect, Moscow 119991, Russia

**Keywords:** sound velocity, pressure drop, acoustics, ultrasonic irradiation, nonlinearity, resonance frequency, extraction, interphase distribution, intensification

## Abstract

The non-linear equation of the radial oscillations of a liquid ball in an immiscible liquid under the exposure of time-varying sound pressure was obtained. The behavioral features of a liquid spherical drop placed in such a media were analyzed in the presence of ultrasound irradiations. The slowing-down effect of the extracted metal ions under its exposure has been studied for the first time, using theoretical and experimental approaches. This phenomenon mechanism was revealed, and analytical equations for the mass transfer rate as a function of the sound pressure oscillations amplitude and the substrate ultrasonic treatment time are presented. Experimental studies of Fe^3+^ ions extracted from chloride and nitrate solutions in systems based on water-soluble polymers were carried out, and a convincing coincidence with the results of theoretical calculations was established. The conditions for achieving the desired extraction efficiency when applying the ultrasonic stimulating effect are specified. The derived result opens the complementary possibility in operations, with the separateness of extraction processes, that which has the essential practical importance.

## 1. Introduction

The liquid extraction of metals is becoming more widespread, increasing its usefulness and undergoing comprehensive modernization and improvement [1,2,3]. This may be illustrated by the use of ultrasound, which apparently appeared as an attempt to improve mechanical mixing with its stimulating effect on the extraction of the substances [4,5,6]. At the same time, efforts to intensify the mass transfer processes that underlie this method inevitably lead in their development to a more detailed consideration of the characteristics of physical and chemical interactions that ensure the extraction efficiency of the extracted compound [7,8,9,10]. As a result, this is accompanied by the discovery of new, interesting phenomena that may have an independent full application. In particular, the undertaken study of the ultrasonic extraction characteristics of iron ions from a droplet located in an immiscible liquid has revealed an anomalous, at first glance, slowing-down effect of the extracted iron ions by ultrasonic (US) irradiation. A similar decrease in the distribution coefficients was also observed in [11] when extracting metal ions (Cu, Cd, Cr, Pb, Ni and Zn) from liquid waste samples. The seeming contradiction arising here with the fixed notion of ultrasound as a means, the use of which is accompanied by an increase in the yield of the final product, or the acceleration of existing interactions, can not only be explained with a detailed analysis of the physical and chemical nature of this phenomenon, but also identify possible areas of its effective introduction into existing chemical and technological processes.

The ultrasound ability to intensify a variety of physical and chemical processes is based on its ability to change certain characteristics of the phenomenon under consideration, and therefore to influence the quantity and quality of the final product in a controlled way. On this basis, a methodology for removing inorganic contamination from soil using ultrasonic cleaning was developed in [12], and in [13] the dependence of sono-chemical reactions on the ultrasonic exposure frequency, based on the characteristics of the cavitation process, was studied. Useful achievements include the successful application of US treatment [14] to increase the adsorption coefficient of various toxic organic dyes and heavy metals by having it deposited onto carbon base nanoparticles. Uses of ultrasonic methods essentially helped to optimize the response surface methodology [15,16] and to find the best conditions for this perspective approach in the liquid-based green extraction. Various manners of using ultrasonics in different chemical applications were described in the valuable survey [17] that can help well to make a good choice while optimizing a large set of chemical processes. However, the serious achievements in the use of ultrasound to intensify the extraction process do not allow asserting the presence of a more or less coherent system of views concerning the scientific understanding of the US interaction processes with ions and molecules during extraction. The authors of [4] paid particular attention to summarizing the results of various works devoted to the use of ultrasound in the liquid membrane method. As was reported in [18], the ultrasound has chemical and mechanical effects on the parameters of reactions, such as increasing the selectivity, enhancing the conversion and rising the yield. It was discovered that ultrasonic energy could break chemical bonds and also initiate some mechanical effects, which are caused by shock waves formed during US cavitation [18]. The present consideration concerns only US intensities, which are less than the threshold value of US cavitations. It permits the avoidance of destructive effects, and to use low-energy sources simultaneously with reaching required results. In some sense, it is similar to the cases investigated in [19], where it has been reported that at low powers, ultrasonic irradiation does not induce any chemical changes, but at a power above some threshold value, expected changes do occur.

In this paper, a specific example of extracting iron ions from dilute solutions, within the framework of an approximate model of the interaction of acoustic flows, arising from ultrasonic pulsations with extraction fluid molecules, was used to study the features of mass transfer at the interface, and to identify the reasons for the slowdown of extraction in the case under consideration. They are related to the spatial structure of the extracted compounds, and can occur when other substances with a similar spatial structure are extracted.

For example, this effect can be of practical importance in the separation of various substances extracted simultaneously by ultrasound. In particular, the deep understanding of the US influence on the velocity of mass transfer processes with metal ions during extraction gives a possibility of increasing the efficiency of the applications of extraction methods to release, separate and clean substances well.

## 2. The Problem Statement and the Initial Ratio

The crushing of immiscible liquids into droplets used for extraction is accompanied by the pulsations of their diameters in the case when the process is influenced by ultrasound. The solution of the problem of the spherical droplet behavior in another immiscible liquid, in which the pressure changes periodically with time *t*, makes it possible to estimate the flows arising in it, and to determine the nature of the oscillations of the droplet with the radius *R*(*t*) in order to calculate the change in its surface area on which the mass transfer occurs. The state of equilibrium between the droplet and the surrounding liquid with a density of ρ_1_(*r,t*) and ρ_2_(*r,t*), respectively, is determined by the balance of all forces acting upon its spherical surface, and can be written as follows [17]:

(1) $$P_{1}(t)=P_{2}(t)+\frac{2\alpha }{R(t)}$$

There *P*_1_(*t*) is the internal pressure of the droplet on its surface, and *P*_2_(*t*) is the corresponding value from the surrounding liquid, while α is the surface tension coefficient.

In the spherical coordinate system with the radial coordinate *r*, the zero value of which is placed in the center of the spherical droplet, the velocity of the radial movement of the liquid *u*(*r*,*t*) inside and outside the globe satisfies the following equations [20,21]:

(2) $$\frac{\partial u}{\partial t}+u\frac{\partial u}{\partial r}=-\frac{1}{\rho }\frac{\partial p}{\partial r}$$

(3) $$\frac{\partial \rho }{\partial t}+\frac{1}{r^{2}}\frac{\partial }{\partial r}\left(r^{2}\rho_{}u\right)=0$$

There *p*(*r,t*) is the local value of the fluid pressure determined by the Tait equation of state [17,18], from which, in the case under consideration, it is possible to write:

(4) $$P_{1}(t)=A_{1}\left\{{\left(\frac{R_{0}}{R(t)}\right)}^{3\gamma }-1\right\}+P_{10}$$

There γ, *A*_1_ are empirically determined constants, and it is assumed that in the initial state the droplet was in equilibrium with the environment, with radius *R*_0_ and a pressure *Ρ*_10_. To calculate the value *P*_2_(*t*), also included in the balance equation (1), it is necessary to solve the system of equations (2)–(3) with the corresponding interface conditions. By introducing the velocity potential φ(*r,t*) (**u** = ∇ φ), the equation (1) can be integrated over the radius r in the range from *r* to ∞, resulting in the ratio:

(5) $$\frac{\partial \phi }{\partial t}+\frac{u^{2}}{2}+\int_{r}^{\infty }\frac{dp}{\rho }=0$$

due to the fact that for *r→∞* there are equals φ = 0, *u* = 0, and the density ρ is associated with the pressure of the Theta equation of state.

The solution of the continuity equation (3) is related to the value of the droplet surface velocity *U*(*t*) = *dR/dt* by the obvious equation which does not take into account the liquid compressibility due to the small contribution to the final result:

(6) $$u(r,t)=\frac{R^{2}(t)U(t)}{r^{2}}$$

Since it is obvious from (6) that φ = −*U R*^2^/*r*, then from (5) for the local pressure *p*(*r,t*) in the liquid surrounding the droplet, the following equation is obtained:

(7) $$\frac{1}{r}\left\{R^{2}\frac{dU}{dt}+2UR\frac{dR}{dt}\right\}-U^{2}R\frac{R^{4}}{2r^{4}}+\frac{1}{\rho_{2}}[P_{\infty }-p(r,t)]=0$$

where *p*(*r→∞, t*) = *P*_∞_(*t*). In the case of a force US action in the liquid, characterized by pressure *P*_m_ sin ωt, with amplitude *P*_m_ and frequency ω, the pressure at infinity (∞) can be set as:

(8) $$P_{\infty }(t)=P_{0}+P_{m}\sin\omega t$$

There *P*_0_ is hydrostatic pressure.

On the droplet surface (*r = R*) the equation (7) with (1) and the obvious relation *P*_10_ = *P*_0_ + 2 α/*R*_0_ can be written as:

(9) $$R\left(t\right)\frac{d^{2}R}{dt^{2}}+\frac{3}{2}{\left(\frac{dR}{dt^{}}\right)}^{2}+\frac{1}{\rho_{2}}\left\{P_{m}\sin\omega t+2\alpha \frac{R_{0}-R(t)}{R(t)R_{0}}+A_{1}\left[1-{\left(\frac{R_{0}}{R(t)}\right)}^{3\gamma }\right]\right\}=0$$

The equation (9) makes it possible to constructively analyze various problems associated with the non-linear evolution in time of the droplet spherical surface from linear pulsations to complex deformation under the US exposure. In particular, the analysis of the intensification characteristics of the mass transfer process in the extractor with the US stimulation system is essentially based on the results of the calculations of the oscillation parameters calculated with the equation (9). It can also be noted that the ratio (9) is analogous to the equation describing the behavior of a gas bubble in liquid [18] under the US exposure, significantly differing from it in the main features, since it is formulated for a different equation of state and other phases.

## 3. Dynamics of Mass Transfer across the Phase Interface under US Exposure

Since the liquid extraction is implemented by means of mass transfer across the phase interface, the change in the conditions of such interaction, for example, the oscillation of the phase interface size, should affect the parameters of this process. A somewhat simplified, but close enough to reality, representation of this process, details can be made on the example of a spherical droplet placed in the immiscible liquid under the sound pressure influence. The radius of such a droplet *R*(*t*), experiencing under the US exposure the oscillations with amplitude δ*R*_m_ and the frequency ω about some equilibrium value *R*_0_, can be described by the following equation 

(10) $$R_{}=R_{0}+\delta R_{m}\sin\omega t+\delta R_{m2}\sin2\omega t+\ldots \ldots$$

The particles transition process of the extracted substance with a characteristic size L from a droplet can be schematically considered as consisting of a stage of the instantaneous transition of the extracted substance particles contacting with the phase interface, after which, at the second stage, the radius decreases by *L*. Then the process is repeated. This model discreteness in the description of the extraction process greatly simplifies the calculations for the evaluation of the most important parameters, but does not significantly affect the accuracy of the calculated final results. In the course of the opposite process of the droplet expansion, the discreteness is provided by a pushing to the particle surface from the inner regions by the radial flows that occur when the external pressure decreases and the droplet size increases. Thus, the model extraction scheme used is based upon the simplifying assumption, that during the change in the droplet radius by the value *L*, a uniform equilibrium distribution of the extracted particles over the entire volume of the droplet is established. It should be emphasized that the use of the concept of particle density distribution in the droplet volume automatically takes into account these specified features of the applied model for calculating the extraction parameters.

**Figure 1:**
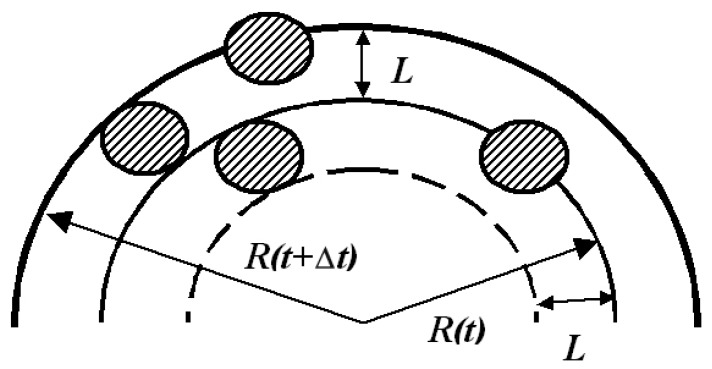
Diagram of the particles extraction process of the extracted substance from a droplet in a single time cycle of the ultrasonic (US) extraction discrete model.

Figure 1 shows the extraction model when the particles of the extracted substance are presented in the form of globes with diameter *L*, as well as the spatial arrangement of these particles, which will be necessarily removed in a single time cycle of the US extraction discrete model. Since in the model under consideration, all particles instantaneously pass from a droplet in contact with or intersecting its surface, then all particles that are completely contained in it, as well as those partially in the near-surface layer, will be removed from the surface layer as it is shown in Figure 1.

The model scheme of the extraction dynamics description under consideration is quite suitable for performing calculations in the case when ultrasound is not used. For the time-independent radius of the droplet, the period Δ*t*, during which the particles fill the near-surface layer, depends on the diffusion flows velocity. In the unsteady case, the particles are introduced into this layer by the flow arising from the pressure difference at the droplet boundary. Therefore, its value L is related to Δt by a simple relation resulting from the representation (10):

(11) $$L=\delta R_{m}\left|\sin\omega (t+\Delta t)-\sin\omega t\right|$$

The equation (11) uses the absolute value of the droplet radius increment for the corresponding time Δ*t*, since the increment itself can have a different sign (droplet expansion or compression), and only the magnitude of this change is of interest. For the region of parameter values ω Δ*t* < 1, in which all practically interesting cases of US extraction are realized, the following estimate of the period Δ*t* of discreteness used in the applied calculation model is obtained from (11):

(12) $$\Delta t≅\frac{L}{\delta R_{m}\omega^{}\left|\cos\omega t\right|}$$

From (12) it can be seen that the discreteness characteristic value of the extraction process in the model under consideration substantially depends upon the value of the time interval between the beginning of the current period of ultrasonic vibrations and the previous act of extracting particles from the droplet. The strict mathematical consideration of this feature of the droplet US pulsation complicates calculations, but does not lead to a significant correction of the final result. Therefore, instead of equation (12), we can approximately use the value averaged over the period of ultrasonic oscillations: *T* = 2π/ω. The approximation results in the following equation instead of (12):

(13) $$\Delta t≅\frac{\pi L}{2\delta R_{m}\omega }$$

The required equation to describe the dynamics of mass transfer can be derived with widely used standard techniques, ranging from the formulation of the basic relations of hydrodynamics [21] to specific laws, for example, changes in the material state in the process of US metals hardening [22]. Let *dq* be the number of extraction cycles that occurred during the time interval *dt* = Δ*tdq*. In this case, the number of particles *dN* extracted from the droplet is determined after the introduction of the concept of the extracted particles density *n*_0_ = 3*N*/(4*nr*_0_^3^) and with the evaluation (13) by the following obvious relation:

(14) $$dN=-4\pi R^{2}(t)Ln_{0}dq\equiv -4\pi R^{2}(t)L\frac{n_{0}}{\Delta t}d\tau$$

Integration (14) results in a final expression describing the time change in the number of particles in the droplet:

(15) $$N(\tau )≅N_{0}\exp\{-6\delta R_{m}\omega \tau /(\pi R_{0})\}$$

It can be seen from (15) that the number of particles decreases exponentially rapidly from the initial value of N_0_ with time, the characteristic period of which depends on the US amplitude and frequency. The nature of this dependence can be determined by solving the following algebraic equations for the amplitudes of the first and second harmonics, which can be derived from (9) with the help of (10) in the form of the equations:

(16) $$\begin{array}{c} \frac{\delta R_{m}}{R_{0}}\frac{\omega^{2}-\Omega^{2}}{\Omega^{2}}=\frac{2\omega^{2}-\Omega^{2}-3\gamma^{2}\Omega_{1}^{2}}{\Omega^{2}}\frac{\delta R_{m}}{R_{0}}\frac{\delta R_{m2}}{R_{0}}-\frac{\left(3\gamma +1)(3\gamma +2\right)}{6}\frac{\Omega_{1}^{2}}{\Omega^{2}}{\left(\frac{\delta R_{m}}{R_{0}}\right)}^{3}+P_{S},\\ \;\frac{\delta R_{m2}}{R_{0}}={\left(\frac{\delta R_{m}}{R_{0}}\right)}^{2}\frac{5\omega^{2}+2\Omega^{2}-\Omega_{1}^{2}(3\gamma -1)}{2(4\omega^{2}-\Omega^{2})}. \end{array}$$

The dimension-free value *P*_S_ in (16) is uniquely determined by the amplitude of the sound pressure *P*_m_, and the symbol Ω is used to indicate the natural frequency of the droplet in accordance with the equations:

(17) $$P_{S}=\frac{P_{m}}{2\rho_{2}R_{0}^{2}\Omega^{2}}{,}_{}^{}\Omega^{2}=\left(3\gamma A_{1}-\frac{2\alpha }{R_{0}}\right)\frac{1}{\rho_{2}R_{0}^{2}}{,}^{}\Omega_{1}^{2}=\frac{3\gamma A_{1}}{\rho_{2}R_{0}^{2}}$$

The exact solution of the cubic equation for δ*R*_m_ is written from (16) as a cumbersome equation, and its analysis is not simpler than the study of the original system (16). However, for practical use it is sufficient to obtain the amplitude δ*R*_m_ of the oscillations in three limiting cases: (1) Ω << ω, (2) Ω >> ω and (3) Ω ≈ ω.

(1) For high value frequencies of external US exposure, when Ω << ω, from (16), (17) follows
  (18) $$\frac{\delta R_{m}}{R_{0}}=P_{S}\frac{\Omega^{2}}{\omega^{2}}$$
(2) In the limit relative to the low-frequency sound signal when the condition Ω >> ω, the solution of the equations system (16), (17) is represented as:
  (19) $$\frac{\delta R_{m}}{R_{0}}=P_{S}$$
(3) In the resonance region Ω ≈ ω, the vibration amplitude of the droplet surface has the maximum value:
  (20) $$\frac{\delta R_{m}}{R_{0}}={\left\{\frac{6P_{S}}{G(\Omega )}\right\}}^{1/3},\;G(\Omega )=(3\gamma +1)(3\gamma +2)\frac{\Omega_{1}^{2}}{\Omega^{2}}-\left(1-3\gamma^{2}\frac{\Omega_{1}^{2}}{\Omega^{2}}\right)\left[7-(3\gamma -1)\frac{\Omega_{1}^{2}}{\Omega^{2}}\right]$$

Thus, substituting the value of the droplet surface vibration amplitude for one of the three possible frequency ranges of external US exposure with equations (18), (19) or (20) in (15), it is possible to trace the dynamics of the US extraction process on the example of the behavior of a single droplet of the liquid phase with the extracted substance. As it was mentioned above, the reason of the stable amount of extracted particles near the boundary might be because of diffusion processes with the velocity *V*_d_ or internal fluxes inside the drop caused by accelerations or slowing down, while meeting with streams of the liquid that may have occurred, for example, because of stirring. In this case, the formula (15) is valid if the velocity 6 δ*R*_m_ω/π caused by ultrasound is substituted by *V*_d_ or by the velocity of stirring.

## 4. Features of Metal Ions Extraction Under the US Exposure

Various extraction liquids are used to extract metal ions from dilute solutions: Organic acids (dialkylphosphoric, dialkylphosphinic, etc.) and their esters (tributylphosphate), salts of quaternary ammonium bases (Aliquate 336), ionic liquids, etc. [23,24,25,26,27]. One of the developing extraction systems for metal ions extraction is that of the two-phase water systems based on water-soluble polymers and inorganic salts [28,29,30].

The extraction of metal ions in systems with polymers depends on the conditions of their extraction, and can be described in a number of cases for the mechanism of its formation of ion pairs due to the electrostatic interaction between protonated polymer and the anionic metal complex [30], which in turn obviously significantly changes the dynamics of the mass transfer through the phases surface interface. In [30] it is shown that the polymer exists in the form of a PEGH^+^ cation in the acidic medium. The metal passes into the polymer phase as the anion HCrO_4_^-^. As a result, both ions charged with opposite signs form a sufficiently strong ion pair [HCrO_4_^−^ PEGH^+^] at the phase interface, which is distributed into the polymer phase.

The electrostatic interaction between the protonated polymer and the anion of the extracted compound is provided by a simple convergence of their centers to a certain limit position, since further convergence is prevented by repulsive forces that begin to dominate at close distances, as it happens, for example, in crystals. As shown in [31], the total electrical potential ψ of two ions with the cubic symmetry of electron shells can be represented as:

(21) $$\psi =-\frac{e^{2}}{\varepsilon_{0}l}+\frac{b}{l^{9}}$$

where *l* is the distance between the ion centers, *b* = const, e is the electron charge, ε_0_ is the electrical permittivity of vacuum. The constant *b* can be expressed in terms of the distance *l*_0_ at which the centers of the approaching ions are in the equilibrium state, when the force F of their interaction vanishes. Since *F* = ∂ψ/∂*l*, this condition results from (21) as a result of differentiation, and leads to the equation:

(22) $$b=\frac{e^{2}}{9\varepsilon_{0}}l_{0}^{8}$$

When ions are removed from the equilibrium position (22), the force of their interaction increases, reaching the maximum value of *F*_max_ at *l* = *l*_m_, and at *l* > *l*_m_, the force *F* decreases with increasing distance. Therefore, the distance of length *l*_m_ = *l*_0_ is the maximum elongation that can withstand the conglomerate in question without being broken. Therefore, the corresponding force value F_m_ is characterized by its tensile strength. The value *l*_m_ can be determined from the condition of existence of the function *F*(*l*) extremum, when the condition ∂F/∂*l*|*_l_*_m_ = 0 should be met, which leads to the equation:

(23) $$\frac{l_{m}^{}}{l_{0}^{}}=\sqrt[{8}]{5}\approx 1,22$$

With (21) and (22) it is also possible to record the formation energy *E*_d_ of the molecular structure from ions:

(24) $$E_{d}\equiv \psi (l_{0})=-\frac{8}{9}\frac{e^{2}}{\varepsilon_{0}l_{0}}$$

The value *E*_d_ from (24) with an inverse sign will respectively represent the dissociation work on the initial ions. The maximum value of power *F*_m_ at the same time has the form 

(25) $$F_{m}=0,68\varepsilon_{0}\frac{E_{d}^{2}}{e^{2}}$$

**Figure 2:**
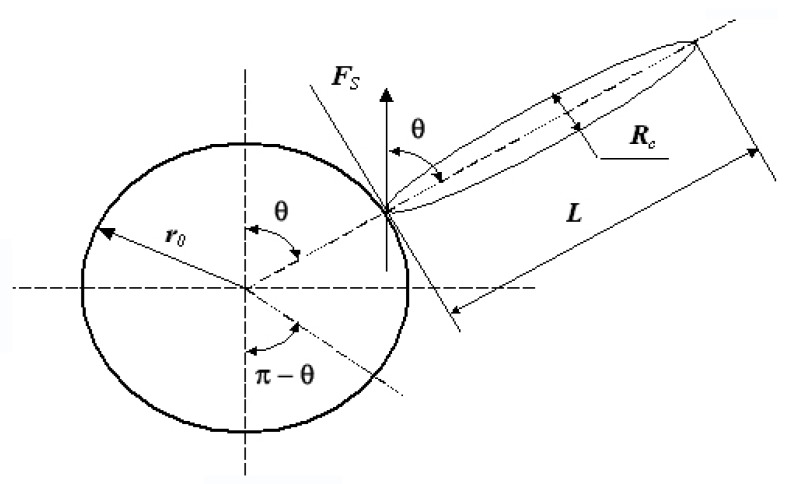
The interaction diagram between the protonated polymer molecule and the anion complex of the extracted metal and the flows inside the oscillating droplet, which allows estimating the conditions of this bond destruction.

These calculations are necessary for the quantitative description of the specific phenomenon accompanying the extraction from the droplets, on which the immiscible liquids are crushed in a wide range of oscillation amplitudes of their radius under the US exposure. The fact is that the rapidly changing in time fluid flows inside the droplet are able to destroy the extractable compound in question, which includes the polymer and anionic metal complex. To obtain the necessary estimates of this process characteristics, one can use its simplified model, in which a negatively-charged anion metal complex is represented as a globe of radius *r*_0_, which is able to attach to a protonized polymer molecule, in turn presented with a good approximation to the cylinder of length *L* and radius *R*_c_. In the liquid phase, such a compound is exposed to radial flows caused by theoscillations of the droplet radius under the US exposure. Schematically, the geometry of this interaction is shown in Figure 2.

The dissociation condition of the considered ionic pair on the initial elements is determined essentially by the balance between the force of electrostatic interaction and the Stokes force [32], acting on their surface when passing round by radial flow. As shown in Figure 2, in order to shift the cylindrical polymer molecule in the direction of the tangent to the globe surface, the corresponding component *F*_S_ sinθ of the action force of the radial fluid flow on it must overcome the force of its interaction with the globe *F*_m_, which is also affected by this flow. 

Therefore, the separation condition is written as a balance of forces acting on the contact area of the surfaces of the protonated polymer and the anion metal complex *F*_m_ ≤ (*F*_SC_ − *F*_SS_) sinθ ≡ *F*_S_ sinθ (26). There, *F*_SC_ is the force acting upon the cylinder of length *L* and radius *R*_c_, which occurs when the flow of a liquid with density ρ, viscosity η and velocity u flows around it, and *F*_SS_ is the same force that appears when the globe with radius r_0_ is flowed around. Equations for these forces are given in [19,22] and have the following form:

(26) $$F_{\mathrm{SS}}=6\pi r_{0}u\eta {;}_{}^{}F_{\mathrm{SC}}=4\pi u\eta L/\ln\{3,7\eta R_{c}/(u_{}\rho )\}$$

The condition (26) defines the cone of angles θ inside which π − θ_0_ ≤ θ ≤ θ_0_ the polymer separates from the globe 

(27) $$\theta_{0}=\mathrm{Arcsin}(F_{m}^{}/F_{S}^{})$$

Polymers, whose active centers are connected to the surface of the spherical particle outside this cone, will retain an electrostatic bond with it, so that such compounds will not be destroyed as a result of the flow impact. Therefore, the relative number of such molecules in relation to their total number will be determined by the ratio of the corresponding surfaces areas. Thus, at the initial value *N*_0_, the total number of extractable compounds from interconnected anion metal complexes and protonated polymers, and the number of intermolecular bonds N_d_ destroyed by the flow, are determined with (28) by the equation:

(28) $$N_{d}=N_{0}\cos\theta_{0}\equiv N_{0}\frac{\sqrt{F_{S}^{2}-F_{m}^{2}}}{F_{S}^{}}$$

The number of remaining intact extracted compounds *N*_S_ formed on the droplet surface–phase interface is calculated by (29) with the following equation:

(29) $$N_{S}=N_{0}-N_{D}=N_{0}\frac{F_{S}^{}-\sqrt{F_{S}^{2}-F_{m}^{2}}}{F_{S}^{}}$$

Thus, in the equation (15), which describes the dynamics of mass transfer across the droplet boundary, instead of *N*_0_, the value *N*_S_ should be used in accordance with its definition (30). It should be noted that over time, after the extraction of the most part of the connected molecules from the droplet, the extraction within the framework of the model under consideration should continue, albeit more slowly, since *N*_S_ will increase due to new compounds of broken structures outside the destruction region (28) of the electrostatic bonds.

It is also necessary to take into account the fact that the flow rate in the Stokes force equation (27) oscillates in time. This means that the period of time when the bonds break under the influence of ultrasound is only a part of its oscillations period, but due to the fact that this rate is included in the pre-exponential factor of the equation (15), taking into account its dependence on time, which is quite a difficult task, this cannot significantly affect the behavior of the curves describing the dynamics of mass transfer. Therefore, in the equation (30) for *N*_S_, an approximate value of the velocity u can be used that replaces its exact value with the amplitude δ*R*_m_ ω.

Equations (15) and (30) allow estimating the change in the concentration of the extracted complex at the stage when rather slow processes of the ions reconnection torn by ultrasound will again combine them into a configuration that will be resistant to external influence, and will lead to the formation of extractable compounds that will be distributed in the polymer phase. As the results of the experiments show, the characteristic time T_A_ of this procedure is very large compared to the period of US oscillation *T* = 2π/ω. It depends on the characteristics of particle collisions in the presence of oscillating fluid flows and other factors, but its introduction makes it possible to generalize the equation (15) to describe the dynamics of the removal of all N_S_ particles from the droplet. Considering that the extraction process of particles remaining after the first stage of US extraction in the amount of *N*_0_ –*N*_d_ begins at the end of time *T*_A_, the equation (15) can be generalized with the method of its derivation by rewriting in the following form:

(30) $$N(\tau )≅N_{S}[1\;-\exp\{-6\delta R_{m}\omega \tau /(\pi R_{0})\}]+0.5N_{d}[1+\mathrm{sign}(\tau -T_{A})][1\;-\exp\{-(\tau -T_{A})/T_{A}\}]$$

Determining the T_A_ dependence on the US parameters and other characteristics of extraction is associated with the solution of a rather complex independent problem, but in the status of the empirical constant, the value *T*_A_ is very useful, as in the description of the mass transfer dynamics in the processes of US metals extraction, as well as for the practical selection of special modes of the corresponding technology operation. For example, this could be when a scheme for the selective extraction of various substances in the case of their simultaneous extraction is being implemented, and the formula (31) can be useful while estimating parameters of extractions and corresponding characteristics of the ultrasound.

The experimental and theoretical results of extraction obtained without ultrasound and under conditions of its application are compared.

The experimental study of the interfacial distribution of Fe^3+^ ions dependence (initial concentration is 0.01 mol/l) from chloride solutions, with the extraction system based on polypropylene glycol 425 (30% (wt.)) and NaCl (8% (wt.)), from time to time with ultrasound, and in its absence, is carried out. The initial solution of iron (III) chloride was prepared by dissolving the sample in a solution of sodium chloride of a given concentration, to which an aqueous solution of polypropylene glycol 425 was then added and stirred for a given time. Further, the phases were separated and the Fe^3+^ ion content was determined in both phases by a spectrophotometric method with sulfosalicylic acid at a wavelength of 420 nm. For the study of the extraction equilibria, the graduated test tubes with ground stoppers were used. The experiments were carried out at the temperature of 25 °C. To ensure a uniform distribution of the components in terms of volume, the mixing was carried out with a 300 rpm upper-drive mixer. To conduct experiments with ultrasound, an ultrasonic generator with a power of 50 W and a frequency of 35 kHz was used. All experimental data presented are the result of a series of experiments, and is processed by mathematical statistics.

**Figure 3:**
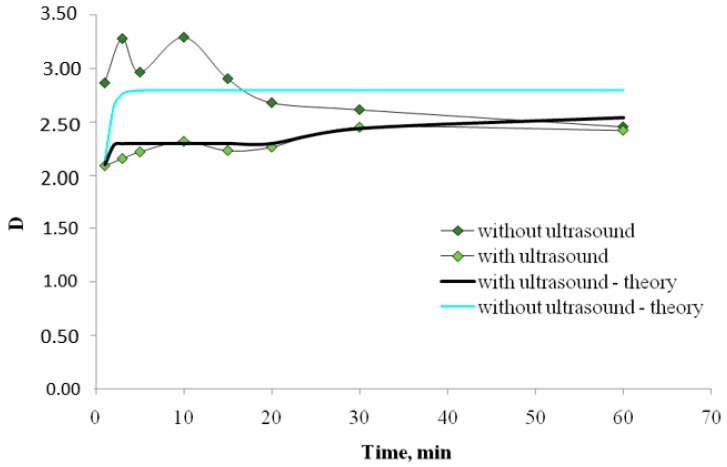
The time dependence of extracted Fe^3+^ ions concentration in the system of polypropylene glycol 425-NaCl-H_2_O under the US exposure (US radiation with the power of 50 W and frequency of 35 kHz), and without ultrasound.

The Figure 3 shows the results of the performed experiments together with the data calculated with the equation (31). Theoretical curves shown in Figure 3, being the function N(τ) in accordance with (31), are obtained for the amplitude of ultrasound *P*_m_ = 0.18 PA, α = 0.075 kg/s, ω = 35 kHz. Calculations of the considered dependence in the absence of ultrasounds were carried out for the magnitude *v*_d_ = 0.4 mm/s. Let us notice that both theoretical curves coincide only for the time of treating *t* > 130 min. It means that the ultrasound is able to slow down extraction processes for a long time. It is important that the functional time dependence of the experimental values of the parameter *N*(τ) corresponds very precisely to the analytical description (31). The good coincidence of these curves indicates the correspondence of the used ideas about the mechanisms of the discussed processes to the reality under consideration.

**Figure 4:**
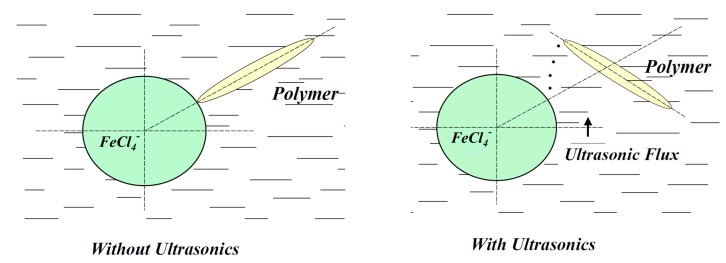
Diagram of the interaction of a protonated polymer molecule with an anionic iron complex under ultrasound conditions and without ultrasound.

The conducted researches of the kinetic dependences of iron ions interphase distribution extracted in the absence and under the US exposure allowed the offering of mechanisms of influence of ultrasonic exposure on extraction, and the defining of possible ways of the practical use of the corresponding effects. Schematically, the proposed mechanism is shown in Figure 4.

The slowing down effect of metals extraction, discussed earlier in the literature [11], has now not only the clear physical explanation and justification, but is provided also with the effective tool for determining process characteristics in the form of simple and convenient equations for carrying out the corresponding calculations on the base of analyzing the details of US interaction with the molecules of the extracted substance. Thus, the detailed study of processes within the spherical drop, that which was carried out elsewhere in [33], where some interesting results of ultrasound uses were generalized on the basis of analyzing enhanced local agitation, induced circulation currents and prolate-oblate oscillations inside the liquid drop, and gives the possibility to use selectively different abilities of ultrasound in solvent extraction systems. The further development of the formulated methods seems to be the most promising, both in the direction of identifying and using the individual characteristics of individual compounds that provide interesting results of US extraction, and in attempts to solve the difficult-to-achieve goals of metal extraction with appropriate US methods, based on the unique possibilities of its interaction with the extraction liquid.

## 5. Conclusion

Based on the results of this work, we can formulate several conclusions summarizing the most important achievements of the research:

1. The analysis of the US deceleration mechanism of metal compounds extraction, performed in this work, shows how the individual features of the spatial configuration of the extracted particles can significantly affect the dynamics of mass transfer in liquid extraction. Under these conditions it is reasonable to assume that a purposeful change in such a configuration can provide a predetermined change in the characteristics of US cleaning technologies. As a result, there are new opportunities for targeted extraction from the introduction of separation schemes to the development of systems for removing difficult-to-extract substances from the initial liquid.

2. The equation obtained in the present work for the radial oscillations of a spherical droplet in an immiscible fluid under sound influence is derived for a realistic Theta equation of state, and can be successfully used, not only in studying the extraction problems, but also for solving problems of intensifying other chemical processes, and changing the technological properties of various systems using US fluid breakup. Analysis of such droplets behavior is also of some interest for the study of a number of physical phenomena accompanying the flotation at surfaces or jet efflux in the liquid.

3. The construction method of simplified (in comparison with reality) models used in this work to describe the parameters of the chemical bonds of molecules, and in the calculation of the dynamic characteristics of extraction, can also be successfully used to predict a number of relevant effects and their design based on the understanding of their functioning mechanisms. For example, some metals and other compounds do not enter into the necessary chemical interaction to ensure the effectiveness of the liquid extraction method. Therefore, it is very attractive to create particles, that by their behavior in a radial oscillating flow under the US exposure, could simply mechanically displace the desired structure from the droplet of the initial liquid without even interacting with it.

4. The revealed good agreement of the experimental results with the calculated data indicates that the real process of US extraction varies in time according to the law, which is in good accordance with the equations obtained from calculations based on a certain representation of the underlying dominant mechanisms. Therefore, this fact proving the validity of the applied theoretical assumptions, can be used in a decisive way to identify and then put into practice other interesting opportunities resulting from the targeted application of ultrasound in extraction technologies.

Thus, the proposed solutions can be used to improve the environmental safety of extraction technologies through the use of energy-efficient ultrasonic exposure, as well as eco-friendly ATPS.
